# The perioperative outcomes of uniport versus two-port and three-port video-assisted thoracoscopic surgery in lung cancer: a systematic review and meta-analysis

**DOI:** 10.1186/s13019-022-02034-y

**Published:** 2022-11-08

**Authors:** Ya-Fu Cheng, Chang-Lun Huang, Wei-Heng Hung, Ching-Yuan Cheng, Bing-Yen Wang

**Affiliations:** 1 Division of Thoracic Surgery, Department of Surgery, Changhua Christian Hospital, No. 135 Nanxiao St., Changhua City, 500 Changhua County Taiwan; 2 School of Medicine, Chung Shan Medical University, Taichung, Taiwan; 3 School of Medicine, College of Medicine, Kaohsiung Medical University, Kaohsiung, Taiwan; 4 Institute of Genomics and Bioinformatics, National Chung Hsing University, Taichung, Taiwan; 5 Department of Post-Baccalaureate Medicine, College of Medicine, National Chung Hsing University, Taichung, Taiwan; 6 Center for General Education, Ming Dao University, Changhua, Taiwan

**Keywords:** Uniport, Single incision, Single port, Video-assisted thoracoscopic surgery, Non-small cell lung cancer

## Abstract

**Background:**

Uniport video-assisted thoracoscopic surgery (VATS) has been applied widely for the treatment of lung cancer in recent years. Some studies have reported that uniport VATS might provide better outcomes than multiport VATS. However, the perioperative outcomes of uniport VATS compared with two-port and three-port VATS, respectively, have yet to be studied at a comprehensive scale. This meta-analysis study compares the perioperative efficacy among uniport, two-port, and three-port VATS.

**Methods:**

We searched studies published before October 1, 2019, by using Web of Science databases, Ovid Medline, Embase, and PubMed. Studies that compared uniport VATS with two-port or three-port VATS for patients with lung cancer were included. Operative time, perioperative blood loss, number of lymph nodes retrieved, conversion rate, duration of postoperative chest tube drainage, length of hospital stay (LoS), visual analogue pain scores on postoperative day (POD) 1 and POD 3, and overall morbidity were evaluated.

**Results:**

Sixteen studies that compared uniport VATS with two-port or three-port VATS in the treatment of lung cancer were included. Uniport VATS showed less blood loss, a shorter duration of postoperative drainage and a lower visual analogue pain score on POD 3 than two-port VATS; it showed a shorter duration of postoperative drainage, a shorter LoS, and lower visual analogue pain scores on POD 1 and POD 3 than three-port VATS. There were no significant differences in the number of lymph nodes retrieved, operative time, conversion rate, and overall morbidity rate when comparing uniport VATS with two-port VATS or three-port VATS.

**Conclusions:**

Uniport VATS might provide better perioperative outcomes than either two-port or three-port VATS in lung cancer treatment.

## Background

Lung cancer is identified as the leading cause of cancer death worldwide in both males and females [1]. Surgery is the treatment of choice for stage I to IIIA non-small cell lung cancer (NSCLC). Thoracoscopic lobectomy for lung cancer was first performed in the 1990s [2]. The advantages of video-assisted thoracoscopic surgery (VATS) include less postoperative pain and a shorter postoperative hospital stay compared with open thoracotomy [3, 4].

Over the past few decades, VATS has been widely applied for diagnosing and resecting lung cancer. VATS most frequently involved two or three operation ports. The number of incisions required for VATS has gradually reduced as instrument and surgical technology has improved over time. Yamamoto et al. first reported six patients with pneumothorax treated with uniport thoracoscopic surgery in 1998 [5]. For complex VATS intervention, Gonzalez et al. introduced the use of uniport thoracoscopic lobectomy in 2011 [6].

The main advantages of uniport VATS include decreased postoperative pain, faster initiation of rehabilitation, reduced hospital stays and improved patient satisfaction [7–9]. There were some studies that compared uniport VATS and multiport VATS among patients with early-stage NSCLC; they claimed that uniport VATS patients had less blood loss and postoperative pain and shorter operative times than multiport VATS patients [10, 11]. However, other studies reported that uniport VATS might provide no significant benefit for perioperative outcomes [12–14]. The perioperative outcomes between different numbers of chest wall incisions remain controversial and have yet to be well studied.

A previous meta-analysis study has reported that uniport VATS provides superior perioperative efficacy than multiport VATS [7]. However, there is no study that separates two-port VATS and three-port VATS in comparisons with uniport VATS. Most experts described two-port VATS as being a bridge from VATS involving three or more ports to uniport VATS. The technical skills and procedure are much easier for two-port VATS compared to uniport VATS. It is still unknown whether uniport VATS provides better perioperative outcomes than both two-port VATS and VATS procedures involving three or more ports.

Thus, we designed this meta-analysis study to compare the perioperative efficacy of uniport, two-port and three-port VATS for patients with lung cancer. We aimed to find out whether uniport VATS leads to better perioperative outcomes than either two-port or three-port VATS. This systematic review and meta-analysis followed the PRISMA statement and guidelines.

## Methods

### Search strategy

We searched for eligible studies by querying Web of Science databases, Ovid Medline, Embase, and PubMed for suitable studies published before October 1, 2019. To identify all relevant studies, we used the following terms as keywords or MeSH terms: (non-small cell lung carcinoma OR lung adenocarcinoma OR lung squamous cell carcinoma OR lung cancer) AND (uniport OR single-port OR single incision OR uniportal). The reference lists of all the qualified papers were inspected for other potentially relevant studies.

### Study inclusion and selection criteria

Both prospective and retrospective studies were eligible. These studies needed to include the perioperative data of patients with lung cancer among uniport and multiport VATS groups. All the included studies needed to observe one of the following features: operative time, perioperative blood loss, number of lymph nodes retrieved, conversion rate, duration of postoperative chest tube drainage, length of hospital stay (LoS), visual analogue score (VAS) of pain for either postoperative day (POD) 1 or POD 3, and overall morbidity. Our exclusion criteria were the following: (I) Studies that compared one-port and multiport VATS but mixed two-port and three-port VATS. (II) Studies that compared one-port and multiport VATS but did not focus on lung cancer treatment. (III) Studies that did not include any relevant data. (IV) Studies that were case reports, case series, review articles, comments or meta-analyses.

The outcome measures included operative time, perioperative blood loss, number of lymph nodes retrieved, conversion rate, duration of postoperative chest tube drainage, LoS, VAS of pain on postoperative days 1 and 3, and overall morbidity rate.

### Data extraction and quality assessment

All the data used in the meta-analysis were extracted from the texts, tables and figures of articles. Two reviewers independently assessed the titles and abstracts of all identified trials to confirm fulfillment of the inclusion criteria (WH Hung and YF Cheng). The following information was collected: author, year, data sources, design of study, surgical method, patient number, operative time, perioperative blood loss, number of lymph nodes retrieved, conversion rate, duration of postoperative chest tube drainage, length of hospital stay, visual analogue score of postoperative pain, and overall morbidity. Any inconsistencies between the two reviewers were resolved by discussion and consensus. The methodological quality of the included studies was assessed by the Newcastle Ottawa Scale (NOS). If any information was unavailable, we contacted the authors through e-mail or telephone to retrieve the data. The final results were reviewed by a senior investigator (BY Wang).

### Statistical analysis

We used Comprehensive meta-analysis (CMA) software version 3.0 (Biostat, Englewood, NJ, USA) to perform the statistical analysis for this study. Between-trial heterogeneity was determined by using I^2^ tests; we interpreted values > 50% as indications of considerable heterogeneity. The meta‑analysis was conducted using a random‑effect model if there was no statistically significant heterogeneity (I^2^ < 50%). Funnel plots were used to examine potential publication bias. All p-values were two-sided. Statistical significance was defined as *p*-value < 0.05. We used the NOS to measure the quality of the studies.

## Results

### Study search and characteristics of included patients

**Fig. 1:**
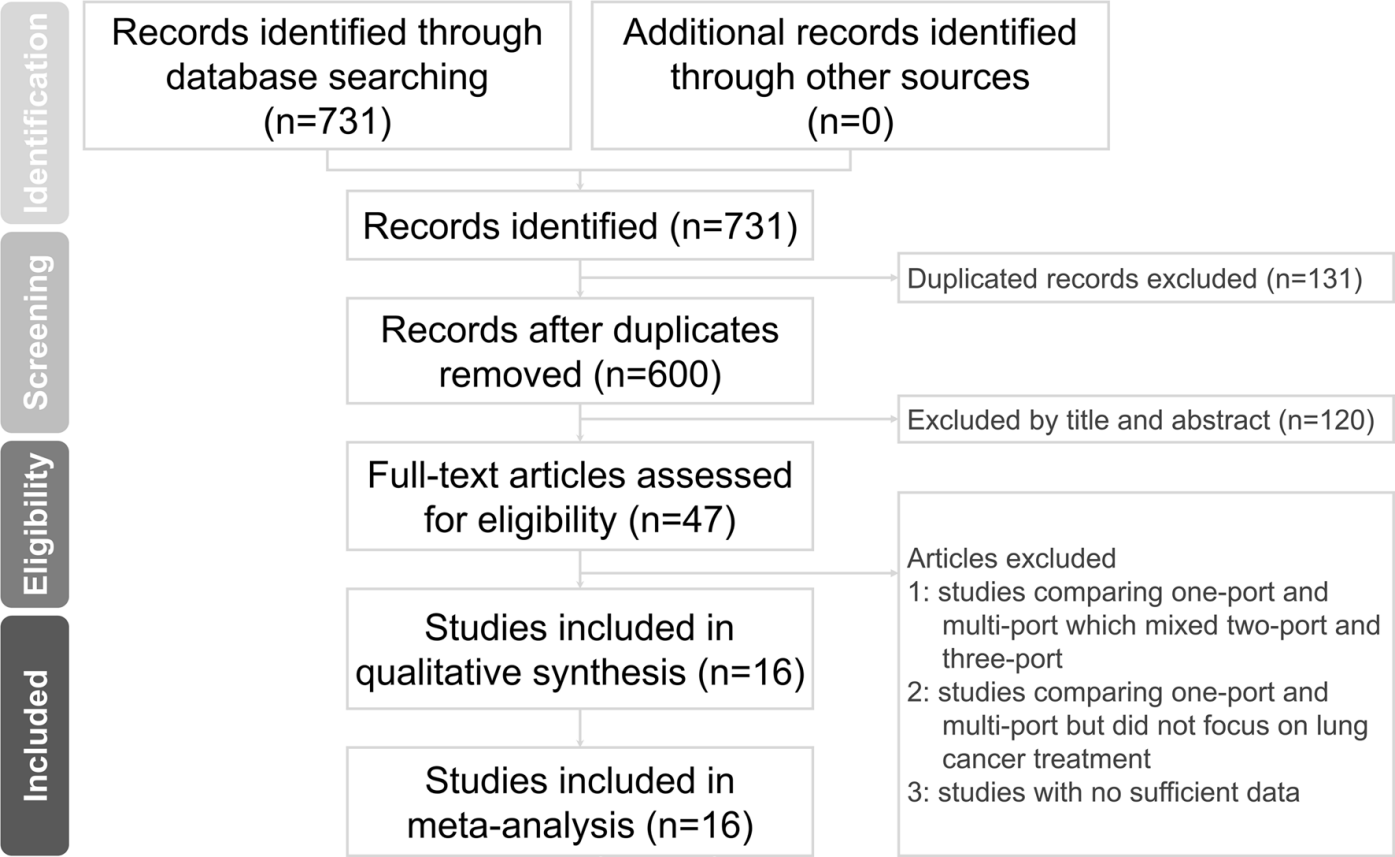
Flow diagram of the study selection process

According to our electronic retrieval strategy, a total of 731 studies were identified. After removing duplicates, irrelevant articles, case reports, reviews, and meta-analyses, the full texts of the remaining studies were scrutinized. After excluding studies that did not provide the appropriate data, sixteen studies were included for final assessment and were deemed to be suitable for quantitative meta-analysis [9, 11–25]. The process of article selection is summarized in Fig. 1.

### Study characteristics and publication bias analysis

Table 1 presents a detailed summary of the study characteristics. Among the sixteen included studies, fifteen were retrospective observational studies, and only one was a prospective cohort study. A total of 3685 patients were included, of which 1428 (38.7%) patients underwent uniport VATS, 296 (8%) patients underwent two-port VATS, and 1961 (53.2%) patients underwent three-port VATS. Seven articles used a propensity score or a matched pair method. All the eligible studies had NOS scores ranging from six to eight.

**Table 1:** Basic characteristics of the publications selected for meta-analysis

Study	Year	Country	Surgery	Trial type	No. of patients			Quality score
					One-port VATS	Two-port VATS	Three-port VATS	
Li C	2013	China	Lobectomy	Retrospective	87		75	6
Zhu Y	2015	China	Lobectomy	Retrospective	33		49	6
Hirai K	2015	Japan	Lobectomy	Retrospective	60		20	6
Mu JW	2015	China	Lobectomy + Segmentectomy + Wedge	Prospective	58		347	8
Shen YX	2015	China	Lobectomy	Retrospective	115		296	8
Chang JM	2016	Taiwan	Lobectomy + Segmentectomy	Retrospective	45	76		6
Hao ZP	2016	China	Lobectomy/Bilobectomy + Pneumonectomy	Retrospective	115		101	6
Dai FQ	2016	China	Lobectomy	Retrospective	77	66		8
Ke HG	2017	China	Lobectomy	Retrospective	65		97	8
Wang LL	2017	China	Lobectomy	Retrospective	73	86	98	6
Heo W	2017	Korea	Lobectomy	Retrospective	37		67	8
Song KS	2017	Korea	Lobectomy	Retrospective	26		47	8
Han KN	2017	Korea	Lobectomy/Bilobectomy + Segmentectomy	Retrospective	203	68	168	6
Liu CC	2018	Taiwan	Lobectomy + Segmentectomy + Wedge	Retrospective	150		382	8
Li C	2018	China	Lobectomy	Retrospective	131		101	6
Wang GX	2018	China	Lobectomy	Retrospective	153		113	6

**Fig. 2:**
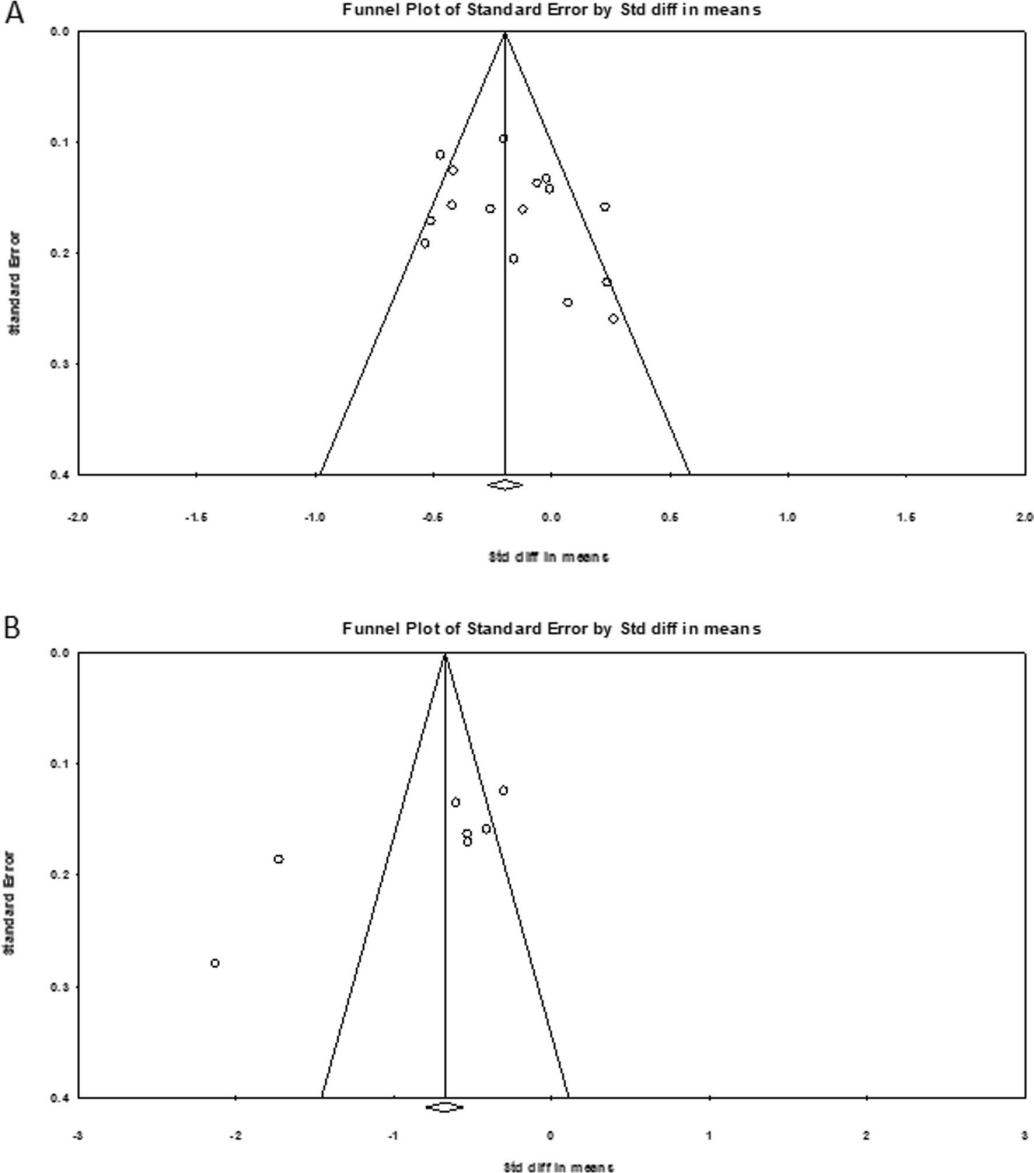
Funnel plots for results included in the meta-analysis: **A** Blood loss of operation, **B** Visual analogue pain score on postoperative day 1

Using Comprehensive meta-analysis Version 3.0, we formed funnel plots to assess publication bias for this meta-analysis. Publication bias was evaluated for the blood loss and visual analogue pain score on POD 1. The results are summarized in Fig. 2; symmetrical funnel plots for perioperative blood loss showed that there was little publication bias in our meta-analysis. However, the funnel plot for visual analogue pain score on POD 1 did not show the satisfying symmetry.

### Operative outcomes

#### Number of lymph nodes retrieved

**Fig. 3:**
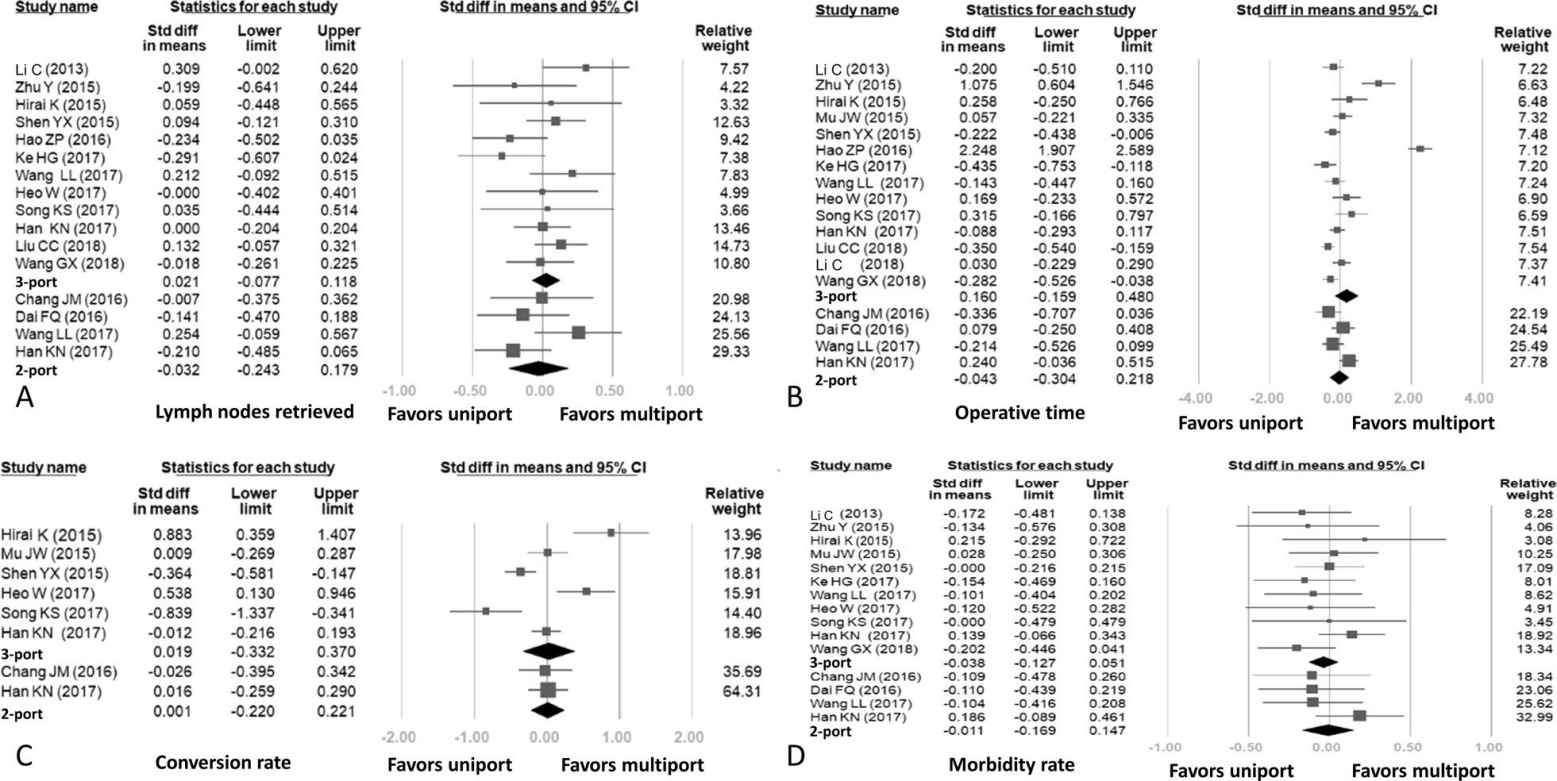
Forest plots for results: **A** Number of lymph nodes retrieved, **B** Operative time, **C** Conversion rate, **D** Morbidity rate

Fourteen studies included data related to the number of lymph nodes retrieved, in which four studies compared two-port VATS with uniport VATS and twelve studies compared three-port VATS with uniport VATS. Thus, two studies involved comparisons of uniport VATS with two-port VATS and three-port VATS, respectively. A combined total of 3048 patients were included. The mean numbers of lymph nodes retrieved for the uniport, two-port, and three-port VATS groups were 18.12 ± 7.6, 18.47 ± 9.2, and 18.13 ± 7.04, respectively; there were no significant differences between the uniport VATS group and the other two VATS groups (uniport vs. three-port: SMD = 0.021, 95% CI − 0.077, 0.118; uniport vs. two-port: SMD = 0.032, 95% CI − 0.243, 0.179). However, low heterogeneity was detected (uniport vs. three-port: *p* = 0.094, I-squared: 40%; uniport vs. two-port: *p* = 0.156, I-squared: 43%). The results of this analysis are given in Fig. 3A.

#### Operative time

Sixteen studies included data related to operative time, in which four studies compared two-port VATS with uniport VATS and fourteen studies compared three-port VATS with uniport VATS. Thus, two studies involved comparisons of uniport VATS with two-port VATS and three-port VATS, respectively. A combined total of 3685 patients were included. The mean operative times for the uniport, two-port, and three-port VATS groups were 154.18 ± 37.9 min, 168.58 ± 48.5 min, and 148.84 ± 45.6 min, respectively. A forest plot suggested that compared with multiport VATS groups, regardless of two or three ports, uniport VATS was not associated with an increased operative time (uniport vs. three-port: SMD = 0.160, 95% CI − 0.159, 0.480; uniport vs. two-port: SMD = − 0.043, 95% CI − 0.304, 0.218). High heterogeneity was detected (uniport vs. three-port: *p* < 0.001, I-squared: 94%; uniport vs. two-port: p = 0.046, I-squared: 63%). The results of this analysis are summarized in Fig. 3B.

#### Conversion rate

Seven studies included data related to the rate of conversion to open thoracotomy or the need for additional ports, in which two studies compared two-port VATS with uniport VATS and six studies compared three-port VATS with uniport VATS. Thus, only one study involved comparisons of uniport VATS with two-port VATS and three-port VATS, respectively. A combined total of 1695 patients were included. The mean conversion rates for the uniport, two-port, and three-port VATS groups were 5.6%, 3.5%, and 4.0%, respectively. There were no significant differences when comparing the uniport VATS group with the other two VATS groups (uniport vs. three-port: SMD = 0.019, 95% CI − 0.332, 0.370; uniport vs. two-port: SMD = 0.001, 95% CI − 0.220, 0.221). High heterogeneity was detected in the uniport versus three-port analysis (uniport vs. three-port: *p* < 0.001, I-squared: 87%; uniport vs. two-port: *p* = 0.858, I-squared: < 0.001%). The results of this analysis are summarized in Fig. 3C.

#### Morbidity rate

Thirteen studies included comparable data related to overall morbidity, in which four studies compared two-port VATS with uniport VATS and eleven studies compared three-port VATS with uniport VATS. Thus, two studies involved comparisons of uniport VATS with two-port VATS and three-port VATS, respectively. A combined total of 2705 patients were included. The mean morbidity rates for the uniport, two-port, and three-port VATS groups were 14.93%, 10.67%, and 18.70%, respectively. Uniport VATS was not associated with a higher rate of morbidity than two-port or three-port VATS (uniport vs. three-port: SMD = − 0.038, 95% CI − 0.127, 0.051; uniport vs. two-port: SMD = − 0.011, 95% CI − 0.169, 0.147). However, low heterogeneity was detected (uniport vs. three-port: *p* = 0.661, I-squared: < 0.001%; uniport vs. two-port: *p* = 0.402, I-squared: < 0.001%). The results of this analysis are summarized in Fig. 3D.

#### Perioperative blood loss

**Fig. 4:**
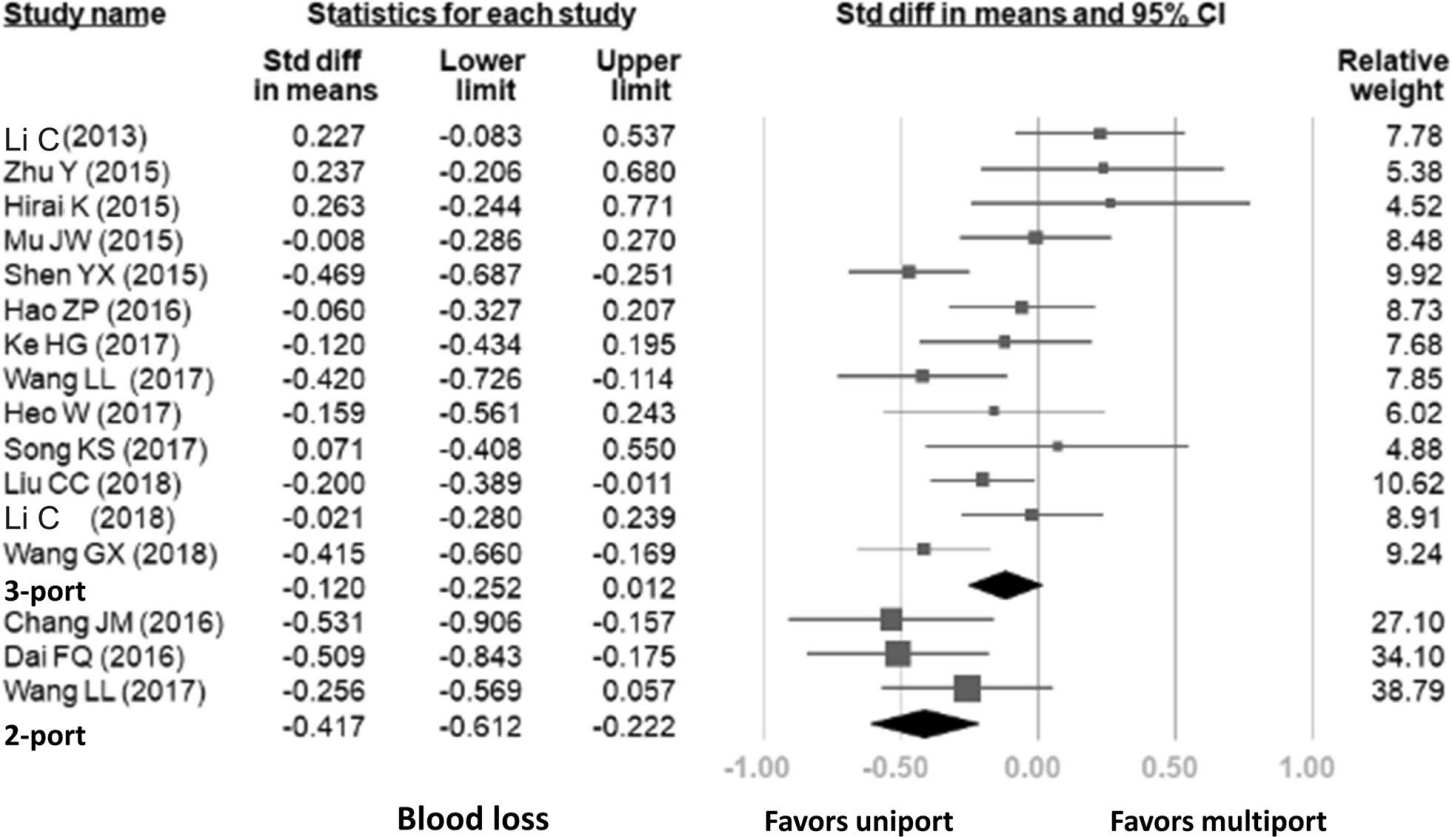
Forest plot of perioperative blood loss for uniport VATS, two-port VATS, and three-port VATS groups

Fifteen studies included comparable data related to blood loss, in which three studies compared two-port VATS with uniport VATS and thirteen studies compared three-port VATS with uniport VATS. Thus, only one study involved comparisons of uniport VATS with two-port VATS and three-port VATS, respectively. A combined total of 3246 patients were included. As shown in Fig. 4, the uniport approach resulted in a significantly lower bleeding volume compared with two-port VATS (SMD = -0.417, 95% CI − 0.612, − 0.222). The uniport technique also seemed to have lower bleeding volume than three-port VATS, but no statistical distinction was observed (SMD = − 0.120, 95% CI − 0.252, 0.012). High heterogeneity was detected in the uniport versus three-port analysis (uniport vs. three-port: *p* = 0.003, I-squared: 60%; uniport vs. two-port: *p* = 0.435, I-squared: < 0.001%).

#### Duration of postoperative chest tube drainage

**Fig. 5:**
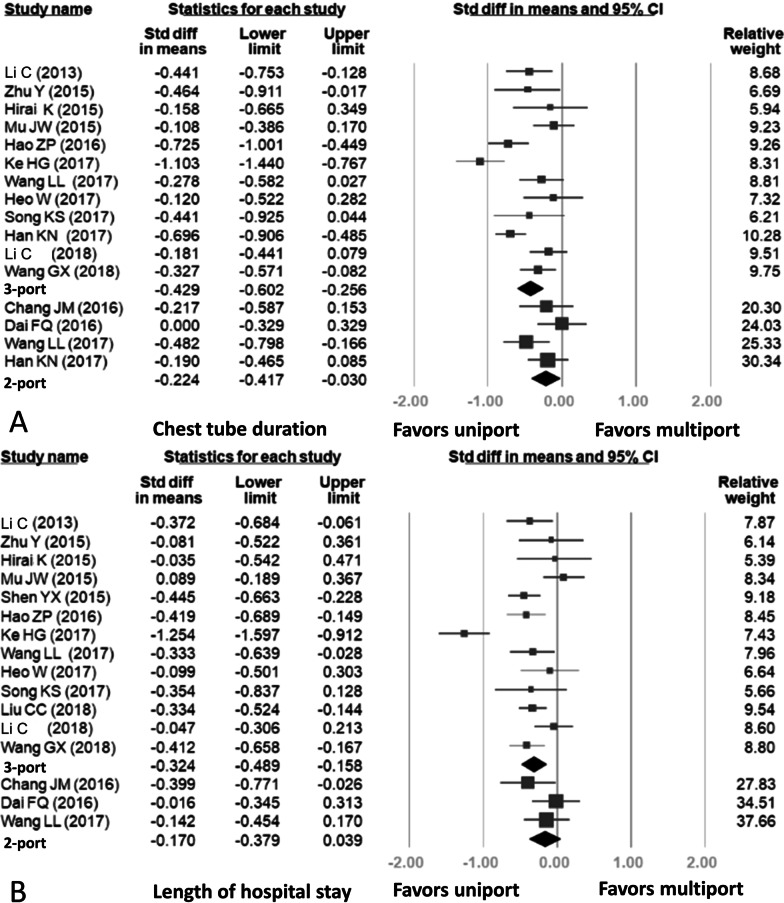
Forest plots for results: **A** Duration of postoperative chest tube drainage, **B** length of hospital stay

Fourteen studies reported data related to the duration of postoperative chest tube drainage, in which four studies compared two-port VATS with uniport VATS and twelve studies compared three-port VATS with uniport VATS. A combined total of 2757 patients were included. The results are summarized in Fig. 5A. The mean chest tube insertion times for the uniport, two-port, and three-port VATS groups were 4.19 ± 2.4, 4.60 ± 2.4, and 5.29 ± 2.7 days, respectively. There was a significant difference between the uniport and two-port VATS groups (SMD = − 0.224, 95% CI: − 0.417, − 0.030). The comparison between the uniport and three-port VATS groups also showed a significant difference (SMD = − 0.429, 95% CI: − 0.602, − 0.256). High heterogeneity was detected in the uniport versus three-port analysis (uniport vs. three-port: *p* < 0.001, I-squared: 72%; uniport vs. two-port: *p* = 0.221, I-squared: 32%).

#### Length of hospital stay

Fifteen studies included comparable data related to length of hospital stay, in which three studies compared two-port VATS with uniport VATS and thirteen studies compared three-port VATS with uniport VATS. Thus, only one study involved comparisons of uniport VATS with two-port VATS and three-port VATS, respectively. A combined total of 2543 patients were included. The results of this analysis are shown in Fig. 5B. The mean lengths of hospital stay for the uniport, two-port, and three-port VATS groups were 7.25 ± 2.9, 8.04 ± 4.0, and 8.36 ± 3.2 days, respectively. There was a significant difference between the uniport and three-port VATS groups but not between the uniport and two-port VATS groups (uniport vs. three-port: SMD = -0.324, 95% CI − 0.489, − 0.158; uniport vs. two-port: SMD = − 0.170, 95% CI − 0.379, 0.039). High heterogeneity was detected in the uniport versus three-port analysis (uniport vs. three-port: *p* < 0.001, I-squared: 74%; uniport vs. two-port: *p* = 0.313, I-squared: 14%).

#### Visual analogue score of pain on postoperative day 1 and postoperative day 3

**Fig. 6:**
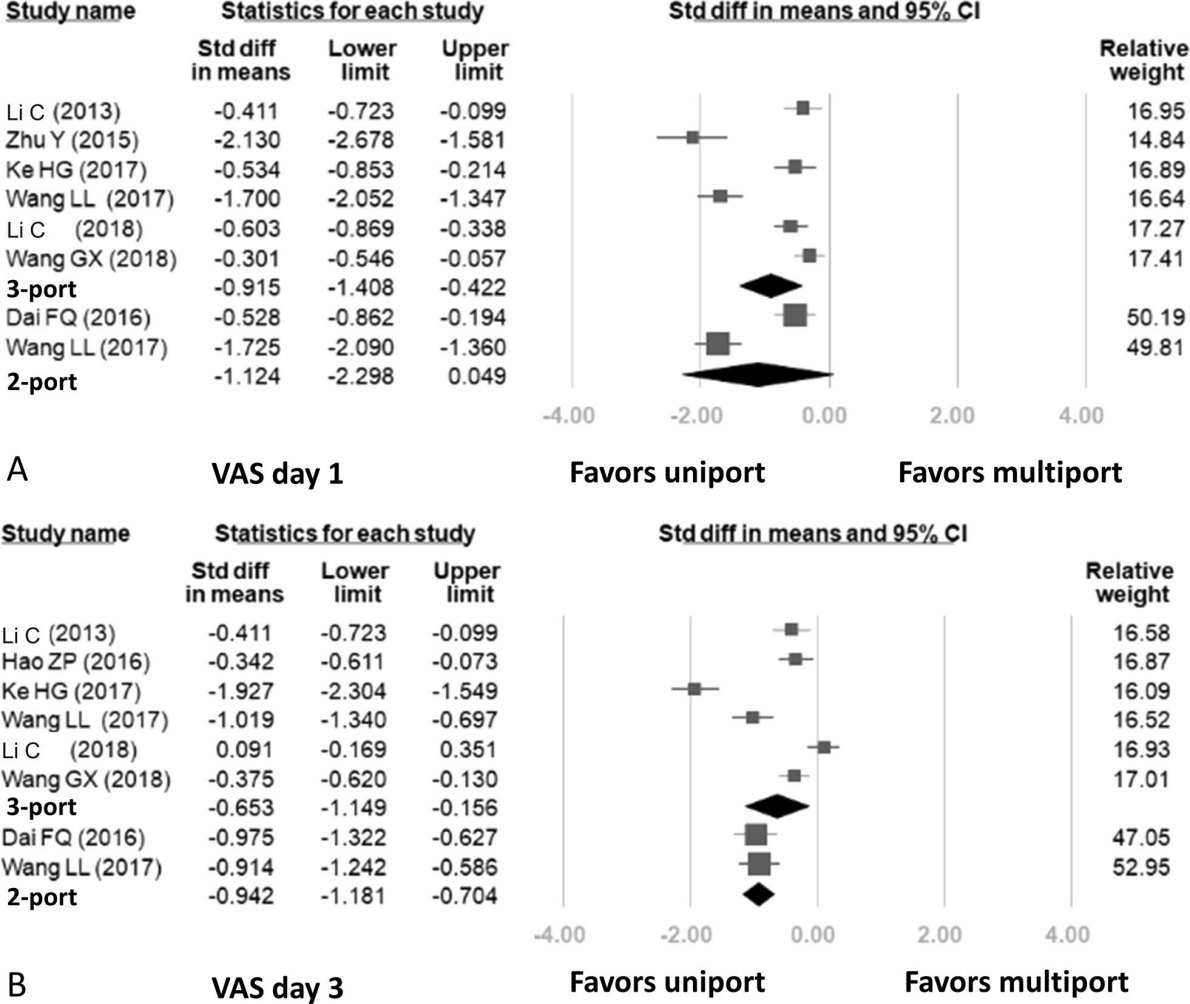
Forest plots for results: **A** Visual analogue score of pain on postoperative day 1, **B** Visual analogue score of pain on postoperative day 3

The results regarding visual analogue score of pain are shown in Fig. 6. Eight studies included data related to pain score, in which two studies compared two-port VATS with uniport VATS and seven studies compared three-port VATS with uniport VATS. Only one study involved comparisons of uniport VATS with two-port VATS and three-port VATS, respectively. A combined total of 1304 patients were included in the analysis of seven studies involving the pain scale on POD 1, and 1518 patients were included in the analysis of seven studies involving the pain scale on POD 3. High heterogeneity was detected regarding the pain scale on POD 1 (uniport vs. three-port: *p* < 0.001, I-squared: 93%; uniport vs. two-port: *p* < 0.001, I-squared: 96%). Forest plots showed that uniport VATS was significantly associated with lower pain on postoperative days 1 and 3 when compared with three-port VATS (VAS day 1: SMD = − 0.915, 95% CI − 1.408, − 0.422; VAS day 3: SMD = − 0.653, 95% CI − 1.149, − 0.156). This trend was also observed in the comparison between the uniport and two-port VATS groups (VAS day 1: SMD = − 1.124, 95% CI − 2.298, − 0.049; VAS day 3: SMD = − 0.942, 95% CI − 1.181, − 0.704).

## Discussion

This meta‑analysis was based on sixteen trials that included 3685 patients. All relevant studies were assessed by the NOS and scored highly. Our meta-analysis demonstrated that uniport VATS was associated with less blood loss, a shorter duration of postoperative drainage and a lower VAS of pain on POD 3 than two-port VATS. Uniport VATS also had lower values than three-port VATS with regard to postoperative drainage duration, LoS, and visual analogue pain scores on POD 1 and POD 3. There were no significant differences in the number of lymph nodes retrieved, operative time, conversion rate, and overall morbidity rate when comparing uniport VATS with two-port VATS or three-port VATS. Our results are consistent with the major findings of previous original studies [7, 26]. Overall, these findings suggest that uniport VATS can be performed with relatively similar or improved perioperative outcomes without compromising safety or oncologic principles.

Uniport VATS allows major thoracic operations to be performed through a single small incision of about 3–5 cm; it can result in less wounding and achieve the same effect as multiport VATS [20]. It eliminates the observation incision at the seventh intercostal space (ICS) and the other working port at the posterior axillary line.

Theoretically, a reduction in the number of incisions is associated with a more difficult surgery. Intense jamming and interference among the thoracoscope and other instruments during uniport VATS were claimed to be the major disadvantages of uniport VATS. Thus, there may be a longer surgical time during uniport VATS [16, 19]. However, our meta-analysis showed that uniport VATS was associated with no difference in operation time. We suggest that surgeons who perform single-port thoracoscopic surgery generally have more experience in VATS. The advanced surgical experience can facilitate less blood loss, improve surgical time and make the surgery more delicate. The learning curve for uniport VATS lobectomy was reported to be thirty cases to reach the performance plateau [27]. After achieving the learning curve, a surgeon can avoid intense jamming of the thoracoscope and other instruments in the limited intercostal space. The number of lymph nodes retrieved, morbidity rate and conversion rate for uniport VATS can also be similar to what is achieved during multiport VATS. Furthermore, uniport VATS can provide the line of vision and operation surface on the same sagittal plane, which makes the axes of thoracoscopic view parallel with the instrumentations. In such a case, the eye-to-hand inconsistency would be lower and the speed of the procedure would be accelerated.

The visual analogue score of pain is a vital indicator affecting the overall treatment effect. It directly affects postoperative performance, rehabilitation, the duration of chest tube drainage and LoS. Our meta-analysis showed that uniport VATS was associated with a lower pain score on postoperative days 1 and 3. The site of incision for uniport thoracoscopic lobectomy is usually located on the 5th ICS along the anterior axillary line. Fewer muscular layers and wider ICS at this area make for less tissue trauma as well. The observation port in the multiport group was usually located at the 7th ICS along the posterior axillary line. The chest wall muscles are thick there and the ICS is narrow, so the blood vessels and nerves could be easily injured. By reducing the number of incisions, tissue trauma of intercostal muscles and nerves can be reduced without increasing the difficulty of surgery. Uniport VATS helps patients to start their rehabilitation earlier and, therefore, accelerates their recovery. [28]

There are few studies that compare the long-term survival rates achieved by the uniport and multiport versions of thoracoscopic surgery for lung cancer. Liu et al. [23] reported that uniport thoracoscopic surgery provided better 5-year overall survival than multiport thoracoscopic surgery in stage I–III lung cancer. Disease-free survival, however, was similar between the two groups. On the contrary, Borro et al. carried out a retrospective review of 276 VATS lobectomies for lung cancer and claimed that the 1- and 4-year survival rates for uniport thoracoscopic surgery were inferior to those for multiport thoracoscopic surgery [29]. Clinical trials are still needed to identify the long-term outcomes between uniport and multiport VATS for lung cancer.

There are several limitations in our systematic review and meta-analysis. Firstly, the lack of long-term survival analysis may lead to deficient oncologic efficacy. Secondly, indirect data acquisition methods were used for meta-analysis, such as the extraction of data from the figures of original articles. Thirdly, all the publications selected for meta-analysis are from East Asia (Japan, Taiwan, China, and Korea); so, this geographical limitation might heavily bias our results. Another limitation is that there is a variability of technique (lobectomy and segmentectomy) included in the selection criteria, although we found that uniport VATS might represent a preferable option for the treatment of T1–3N0M0 NSCLC. Fourthly, heterogeneity was observed in some outcomes (pain VAS on POD 1). Finally, most of the studies involved in our analyses were retrospective; there is a possibility of selection bias and observer bias with regard to the adoption of the operative approach. This may lead to less powerful results, although seven studies performed propensity‑matched analyses to improve the matching of patients according to relevant prognostic factors. More prospective studies and randomized controlled trials are needed.

## Conclusions

Compared to two-port and three-port VATS approaches, uniport VATS might be a superior option for the treatment of early-stage lung cancer. Our meta-analysis provided evidence that uniport VATS could have similar or better perioperative outcomes without compromising safety or oncologic principles.

## Data Availability

The datasets used and analyzed during the current study are available from the corresponding author on reasonable request.

## Abbreviations

NSCLC: Non-small cell lung cancer
VATS: Video-assisted thoracoscopic surgery
LoS: Length of hospital stay
VAS: Visual analogue score
POD: Postoperative day
NOS: Newcastle Ottawa Scale
CMA: Comprehensive meta-analysis
